# Inequivalent and uncorrelated response priming in motor imagery and execution

**DOI:** 10.3389/fpsyg.2024.1363495

**Published:** 2024-05-27

**Authors:** Hsin-Ping Tien, Erik C. Chang

**Affiliations:** ^1^Action and Cognition Laboratory, Institute of Cognitive Neuroscience, College of Health Sciences and Technology, National Central University, Taoyuan, Taiwan; ^2^Taiwan International Graduate Program in Interdisciplinary Neuroscience, National Central University and Academia Sinica, Taipei, Taiwan

**Keywords:** motor imagery, functional equivalence hypothesis, motor simulation theory, S-R binding, repetition and binding

## Abstract

**Introduction:**

Theoretical considerations on motor imagery and motor execution have long been dominated by the functional equivalence view. Previous empirical works comparing these two modes of actions, however, have largely relied on subjective judgments on the imagery process, which may be exposed to various biases. The current study aims to re-examine the commonality and distinguishable aspects of motor imagery and execution via a response repetition paradigm. This framework aims to offer an alternative approach devoid of self-reporting, opening the opportunity for less subjective evaluation of the disparities and correlations between motor imagery and motor execution.

**Methods:**

Participants performed manual speeded-choice on prime-probe pairs in each trial under three conditions distinguished by the modes of response on the prime: mere observation (Perceptual), imagining response (Imagery), and actual responses (Execution). Responses to the following probe were all actual execution of button press. While Experiment 1 compared the basic repetition effects in the three prime conditions, Experiment 2 extended the prime duration to enhance the quality of MI and monitored electromyography (EMG) for excluding prime imagery with muscle activities to enhance specificity of the underlying mechanism.

**Results:**

In Experiment 1, there was no significant repetition effect after mere observation. However, significant repetition effects were observed in both imagery and execution conditions, respectively, which were also significantly correlated. In Experiment 2, trials with excessive EMG activities were excluded before further statistical analysis. A consistent repetition effect pattern in both Imagery and Execution but not the Perception condition. Now the correlation between Imagery and Execution conditions were not significant.

**Conclusion:**

Findings from the current study provide a novel application of a classical paradigm, aiming to minimize the subjectivity inherent in imagery assessments while examining the relationship between motor imagery and motor execution. By highlighting differences and the absence of correlation in repetition effects, the study challenges the functional equivalence hypothesis of imagery and execution. Motor representations of imagery and execution, when measured without subjective judgments, appear to be more distinguishable than traditionally thought. Future studies may examine the neural underpinnings of the response repetition paradigm to further elucidating the common and separable aspects of these two modes of action.

## 1 Introduction

The concept of motor imagery (MI), which involves mentally simulating body movements without any physically overt motor output, has long been linked to motor execution (ME; Jeannerod, 1995; O’Shea and Moran, 2017). Johnson (1982) initially illustrated the interference effect between MI and ME when both were disrupted by alternative tracking tasks. Since then, numerous studies have consistently highlighted the parallel characteristics of MI and ME, both in behavioral performance and the corresponding brain regions.

Evidence supporting the equivalence of MI and ME is found in various behavioral tasks. For example, studies involving drawing and writing tasks have shown that participants exhibit comparable task durations when merely imagining or actually performing the task (Decety et al., 1989). Moreover, a linear relationship exists between mental and physical efforts and autonomic responses, such as heart rate and pulmonary ventilation, during imagined or actual locomotion tasks (Decety et al., 1991). Notably, MI training has proven effective in enhancing performance in sports (Morone et al., 2022; Lindsay et al., 2023), surgical skills (Sapien and Rogers, 2010; Goble et al., 2021), muscle strength (Sidaway and Trzaska, 2005; Paravlic et al., 2018), and even improving motor function in individuals with various movement deficits, such as those with cerebral palsy (Cabral-Sequeira et al., 2016; Gentile et al., 2024), post-stroke patients (Page et al., 2001; Monteiro et al., 2021), and Parkinson’s disease patients (Tamir et al., 2007; Caligiore et al., 2017).

In addition to behavioral studies, neuroimaging research has identified overlapping brain regions for MI and ME, which include the primary motor cortex (M1; Munzert et al., 2009), premotor cortex (PMC; Gerardin et al., 2000; Hétu et al., 2013; Ridderinkhof and Brass, 2015), supplementary motor cortex (SMC; Lotze et al., 1999), cerebellum (Gerardin et al., 2000; Ridderinkhof and Brass, 2015), basal ganglia (Gerardin et al., 2000; Ridderinkhof and Brass, 2015), and posterior parietal cortex (PPC; Ridderinkhof and Brass, 2015). Electrophysiological data also reveal common motor-related sources in both MI and ME, characterized by event-related desynchronization (ERD) in the alpha band during cue-driven motor tasks (Pfurtscheller and Neuper, 1997; Brinkman et al., 2014; Duann and Chiou, 2016), as well as similar beta suppression in both modalities (Burianová et al., 2013). These findings support the notion of functional equivalence between ME and MI and provide insights into how MI can influence subsequent motor performance.

However, studying MI presents substantial challenges due to its covert nature, as it requires participants to construct mental scenarios without performing any observable actions. This often forces MI studies to depend on participants’ self-reports in which they make conscious and subjective claims directly about the mental scenarios, leading to various complexities and potential inconsistencies in the research outcomes. One commonly used measure in MI research is the reported duration of MI (Guillot et al., 2012). Participants are instructed to mark the onset and offset of both imagined and actual actions, often by using a stopwatch in what is known as the mental chronometry approach (Posner, 2005). The time interval between these timestamps serves as the operational definition of performance duration, demonstrating reliability in accessing MI (Malouin et al., 2008). However, conclusions drawn from this “subjective” paradigm have yielded inconsistent results (Guillot et al., 2012). Some studies have reported equivalence between MI and ME in tasks involving gross motor control (arm movement: Gentili et al., 2004, walking: Papaxanthis et al., 2003) and fine motor control (drawing and writing: 4), Conversely, other studies have found the opposite pattern (Reed, 2002; Calmels et al., 2006; Louis et al., 2011). For example, biases in overestimating short durations and underestimating long durations (Wearden, 2003) have been found when estimating duration for MI compared to ME (Calmels et al., 2006). Additionally, durations of MI and ME for the same action are equivalent in aroused but not relaxed state (Louis et al., 2011).

The discrepancies observed can be partly attributed to biases in time perception. Studies have shown that when estimating durations for MI compared to ME, participants tend to overestimate shorter periods and underestimate longer ones (Wearden, 2003; Calmels et al., 2006). This indicates that differences in how MI and ME are processed over time may be significant. Furthermore, the necessity to switch tasks between reporting start and end times and engaging in imagery could delay the process, leading to the overestimation of brief durations. On the other hand, a lapse in attention during tasks that require longer durations might disrupt the imagery process, causing an underestimation in subjective time assessments.

The complexity in understanding the subjective duration of MI in research is further compounded by the demands of various tasks, which can create misleading perceptions influenced by previous experiences. This could potentially skew the perceived relationship between the subjective durations of MI and ME (Decety et al., 1989). Furthermore, explicit verbal knowledge of a motor skill has been shown to disrupt the smoothness of MI and increase its duration relative to ME (Reed, 2002).

In summary, the perceived duration of imagery in MI studies is subject to several influencing factors, including attentional dynamics such as task switching, attentional states, and the modulation of knowledge. These elements, although not directly related to motor control, can significantly impact interpretations of MI and its association with ME. It is crucial for researchers to meticulously acknowledge and mitigate these confounding variables in their investigations of MI to ensure more accurate and reliable conclusions.

The core aim of the present study is to introduce a less subjective method for evaluating the behavioral characteristics of MI through the application of the repetition effect. This effect, a well-documented phenomenon in motor control research, is known for creating short-term stimulus–response bindings across consecutive trials (Bertelson, 1965; Henson et al., 2014). Such binding, when formed in a given trial (n), results in quicker reaction times when overlapped stimulus and response features are encountered in the following trial (n + 1). Unlike the traditional reliance on measuring reported movement duration, the repetition effect provides a less subjective approach to assess the MI process by examining its influence on subsequent motor performance. This paradigm shifts toward using the repetition effect offers a novel way to reduce subjectivity while studying MI, focusing on measurable behavioral changes like reaction time, thereby circumventing the limitations of subjective self-reports.

The phenomenon of “action priming,” characterized by the repetition effect, has been previously observed in MI (Li et al., 2005; Ramsey et al., 2010; Toovey et al., 2021). However, there have been concerns regarding the methodology employed in some of these studies. For instance, Toovey et al. (2021) reported repetition benefits in both MI and motor preparation, but these benefits may have been inflated due to the greater number of repeated prime-probe pairs in their experiment. Additionally, the presentation of prime and probe stimuli in either the left or right visual field introduced the influence of spatial congruency, potentially complicating the outcomes due to attentional orientation (Posner, 1980). In light of these concerns, the present study seeks to address these methodological issues in order to provide a more accurate and less subjective assessment of the behavioral characteristics of MI.

Besides the methodological issues, the inhibitory processes in motor imagery are distinct from the facilitation effects often seen in action priming studies. Studies, including those by Rieger et al. (2017), Bart et al. (2021), and Scheil and Liefooghe (2018), show that MI can lead to slower movement reaction times and both global and effector-specific inhibition, particularly when the same effector is involved in both imagined and executed actions. However, these inhibitory after-effects might be influenced by experimental instructions requiring participants to indicate imagined movement onset and offset, potentially leading to higher effector activity just before MI and introducing inhibitory effects.

Rieger et al. (2017) suggest that inhibition may be coded into the stimulus during MI, affecting action repetition. This effect could be influenced by the experimental setup, especially when visual cues intertwine MI and ME without prior preparation, as noted by Verbruggen et al. (2008). In such cases, the inhibitory process following the visual cue during MI could strengthen the stimulus-inhibition binding. In experiments where participants alternate between MI and ME, uncertainty about the response type can induce reactive inhibitory control, similar to the Go/No-Go paradigm (Rieger and Gauggel, 1999; Verbruggen et al., 2008). However, in practical MI applications, participants are typically well-prepared not to transmit motor commands during MI (Guillot et al., 2012), suggesting that the context and intention in MI significantly influence the nature of inhibitory processes.

The current study aims to verify the theoretical perspective from Jeannerod (1995), which posits similar causal roles and representations between MI and ME. To this end, we employ the “repetition effect paradigm,” a less subjective behavioral approach, responding to previous inconsistencies noted in action priming research (Rieger et al., 2017; Toovey et al., 2021).

One key concern being addressed is the potential boosting of repetition benefits due to unbalanced ratios and attentional orientation. Additionally, the study aims to explore the possibility that repetition costs may be attributed to stronger suppression induced by the measurement process and reactive inhibition. The study takes measures to minimize the influence of attentional factors by avoiding spatial orienting in the central stimuli.

To investigate the nature of MI and its preparatory inhibition, the current study employs a block-design approach. In this design, repetition effects are probed using pairs of imagery-execution or execution-execution trials. By separating MI and ME trials into distinct blocks, participants are made aware in advance whether they should suppress their responses or not. This design helps reveal the pure influence of MI on subsequent execution.

Furthermore, the study employs a speeded-choice task that requires simple key presses in response to central primes and probes. This task is intentionally simpler than those used in previous MI studies, as it eliminates the need for coordination among multiple joints. The aim is to minimize uncontrollable covert attention switching between motor effectors and simplify the motor processing as much as possible.

Based on the functional equivalence hypothesis positing that MI and ME share similar representations and mechanisms, the current study expects the following outcomes:

- *Significant repetition effects in both MI and ME:* This hypothesis is grounded in the idea that both imagined and executed motor actions engage similar neural and cognitive processes. As the repetition effect suggests that repeating a motor task can lead to faster and more efficient processing, observing significant repetition effects in both MI and ME conditions would support the notion that imagining an action and physically performing it involve overlapping cognitive and neural mechanisms. This hypothesis is consistent with theories that posit MI as a functional equivalent to ME in terms of motor planning and execution processes.
- *An equivalent magnitude of repetition effect in MI and ME*: This hypothesis extends the first by positing not only that repetition effects will be observed in both MI and ME but also that these effects will be of comparable magnitude. This is an important distinction, as it suggests a quantitative equivalence in how MI and ME influence motor processing. If the magnitude of repetition effects is similar across both conditions, it would provide stronger evidence for the functional equivalence hypothesis, indicating that MI and ME may exert similar levels of influence on motor system priming and readiness.
- *A significant correlation between the repetition effects observed in MI and ME*: The third hypothesis investigates the relationship between the repetition effects observed in MI and ME. A strong correlation would indicate that individuals who exhibit more pronounced repetition effects in ME are likely to show similar effects in MI, and vice versa. This correlation would provide evidence for individual differences in the capacity for motor simulation and execution, suggesting that the cognitive and neural mechanisms underlying MI and ME are not only similar but also interconnected. It would further imply that the ability to effectively engage in MI could be predictive of performance in ME, which could have significant implications for training and rehabilitation programs that utilize MI techniques.

Experiment 1 introduces the repetition priming paradigm to test the functional equivalence of MI and ME. Experiment 2 addresses potential confounders identified in Experiment 1, employing electromyography (EMG) to monitor and control for subthreshold muscle contractions during MI. Additionally, we extend the duration of prime responses to enhance the quality of the imagery process. These refinements aim to yield more reliable and insightful data on the MI-ME relationship.

## 2 Experiment 1: comparing repetition effects in MI and ME

In this experiment, we employed a forced-choice key-pressing task where the critical independent variables were the identification of prime and probe cues (i.e., repeated and non-repeated) and the type of prime response (i.e., passive observation, mental imagery of movement, or physical execution). The dependent variable of interest was the response time (RT) of the imperative probe response, which serves as an indicator of the after-effects of the prime response when comparing repeated and non-repeated conditions.

Previous research has established that the motor programming process during MI can contribute to the S-R binding (Ito, 1999; Liefooghe et al., 2021). Furthermore, if motor representations are constructed, the S-R binding should elicit facilitation, aligning with the findings of Bertelson (1965). In line with the theoretical framework proposed by Jeannerod (1995), we anticipate observing a repetition facilitation for MI that is equivalent to ME, as opposed to the absence of a facilitation effect associated with mere observation of the stimulus. Furthermore, we hypothesize a correlation between the repetition effects in MI and those observed in ME, suggesting a parallel in the way the brain processes these two modalities of motor planning.

### 2.1 Materials and methods

#### 2.1.1 Participants

We recruited a total of 32 participants (8 males/24 females) whose ages ranging from 20 to 24 years (M = 21.34 years). Participants were from the student population of National Central University, Taiwan, and were screened to exclude known psychotic disorders or visual perception problems. All participants were strongly right-handed, as indicated by a mean score of 90.6 ± 12.5 on the Edinburgh Handedness Inventory - Short Form (Veale, 2014). The study protocol was approved by the Research Ethics Committee of National Taiwan University, and all participants provided informed consent after receiving a comprehensive explanation of their rights and the study procedures.

To determine the appropriate sample size for our study, we initially analyzed data from the first six participants who each completed a set of counterbalanced trials for every color-response pairing. Utilizing G*Power (Faul et al., 2007) we aimed for an effect size of Cohen’s *d* = 0.42. This effect size was based on the observed difference between repeated and non-repeated trials specifically within the MI condition. The ideal power was set at 0.80, and the program estimated that 32 participants would be sufficient to reach an actual power of 0.80.

#### 2.1.2 Task, stimuli, and apparatus

For response input, participants used a mechanical gaming keyboard (model MEKA G1, Thermaltake Esports, Taiwan) with a polling rate of 1,000 Hz. The task was displayed on a 23” LED flat panel monitor (AOC I2379VHE) with a vertical retrace rate of 60 Hz. The stimuli consisted of square boxes, each extending 1.5 degrees within the visual angle, presented against a gray background.

**Figure 1:**
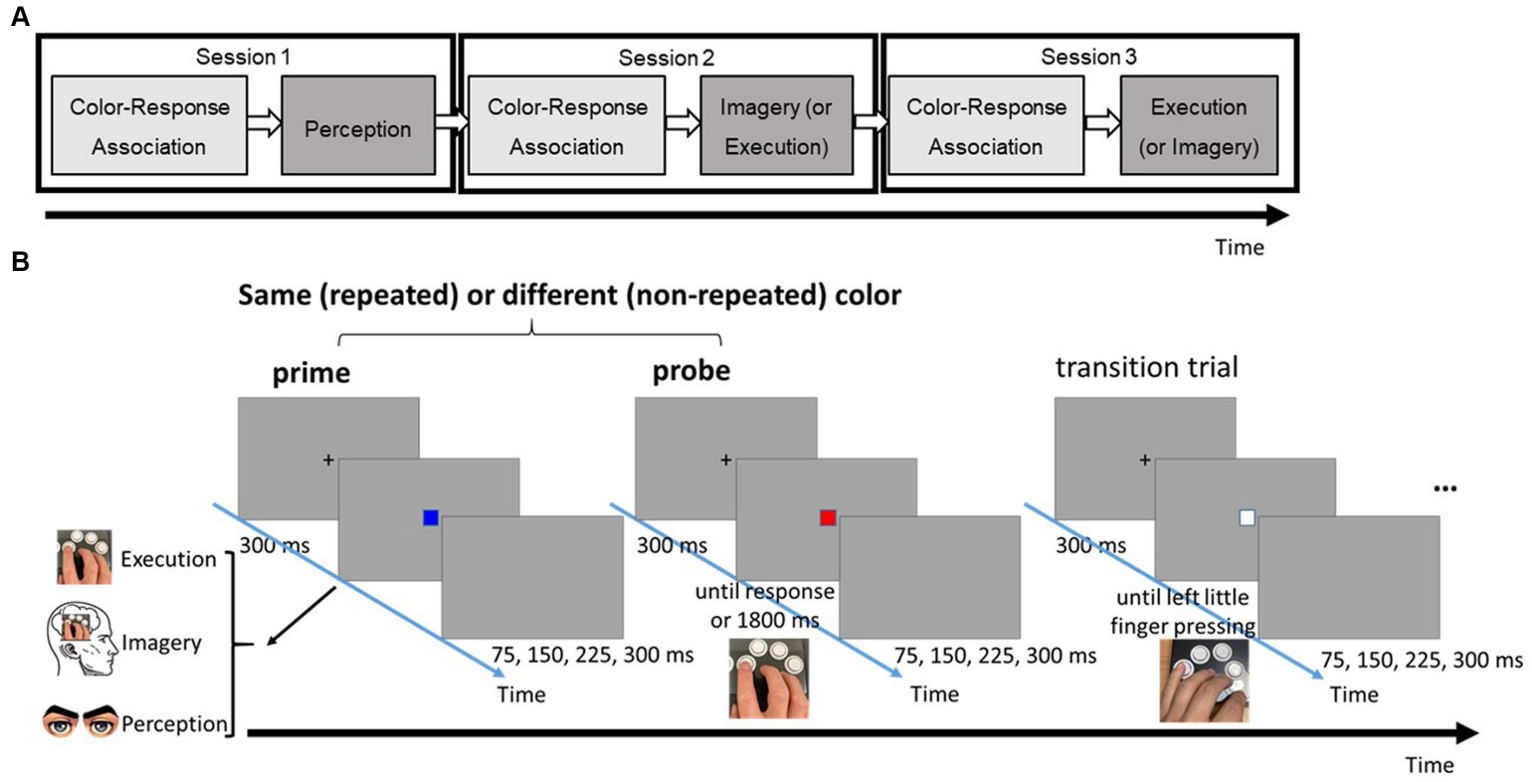
**(A)** The flow of three different task sessions. **(B)** Series of events in a typical trial of the three-choice, speeded response task in the testing phase. Note that there were three possible types of prime: perception, imagery, and execution; each requires a different type of response.

The primary task was a three-choice speeded response task, comprising two phases in each condition session (see Figure 1A). The color-response association phase followed a conventional speed-choice task format, including feedback. Subsequently, the main testing phase utilized a modified speed-choice task without feedback. Each trial in the main testing phase featured two target boxes, each of which could be one of three possible colors (i.e., red, blue, or green). These target boxes were separated by a transition box in white (see Figure 1B). Participants were given explicit instructions corresponding to different priming conditions (Perception, Imagery, or Execution) for responding to a first box (prime). Following their prime response, participants were then required to make a physical key response to a second box (probe). The specific key response for the box was imperatively determined by the color presented.

To ensure a reset between trial pairs and minimize the after-effect of the preceding trial pair, a transition box was inserted. Participants were required to physically press a key in response to the transition box using their left little finger, a finger not involved in the prime and probe responses. Throughout the task, participants were instructed to respond with both speed and accuracy.

#### 2.1.3 Design

There were two independent variables: Prime Type (Execution/ Imagery/ Perception) and Repetition (Repeated/ Non-repeated), and both were within-subject. The dependent variable was the reaction time (RT) to the probe.

To reduce potential uncertainty in prime response and mitigate any reactive inhibition elicited by the Imagery cue, trials with the same prime condition were clustered together and conducted in distinct sessions. As a result, there were three separate sessions, each corresponding to one of the prime conditions: Perception, Imagery, and Execution.

In each session, a combination of 1:2 repeated and non-repeated trials was randomly intermixed to establish a balanced and unbiased distribution of predictability for the specific prime color. This randomization strategy was implemented to control for potential sequencing effects and uphold the integrity of the experimental design.

#### 2.1.4 Procedure

Figure 1A provides an overview of the task flow in the current experiment. Participants completed three distinct sessions, each corresponding to one of the three prime conditions. To prevent automatic motor responses to prime stimuli based on prior experience, the Perception condition always assigned as the first session. The order of the Imagery and Execution conditions was counterbalanced across participants. According to insights from our pilot study, conducting Perception in all three sessions resulted in no systematic changes in the distinction between probe responses after observing the same stimulus vs. different ones across the sequence of sessions (*F*(5, 55) = 0.39, *MSE = 0.00*, *p* = 0.85). Had the Imagery or Execution condition been the initial session, there would have been a greater likelihood of participants’ responses in the subsequent Perception condition being implicitly influenced by motor processes triggered from prior experience in responding to the prime boxes, even if they were instructed otherwise.

During the experiment, participants were seated comfortably in front of a laptop, maintaining a constant distance of 49 cm between their eyes and the screen using a chin-rest. They executed the task by positioning their right index, middle, and ring fingers, along with their left little finger, on the “←,” “↓,” “→,” and “z” keys, respectively. Each session encompassed two distinct phases: the color-response association phase and the testing phase.

The color-response association phase aimed to establish the visuomotor association prior to the testing phase. In this phase, participants familiarized themselves with the relationship between colors and the corresponding fingers (e.g., “red”-“index finger,” “blue”-“middle finger,” and “green”-“ring finger”). Each trial in this phase commenced with a fixation cross displayed at the center of the screen for 300 ms. Immediately following the fixation cross, a colored box was presented and disappeared upon the participant’s response or after an 800 ms time limit. Visual feedback, such as “correct key,” “wrong key,” or “please press the key before the box disappears” was provided on the display following each response. The screen then remained blank for 1,000 ms as participants prepared for the next trial. After completing 12 consecutive trials, participants could take a break until they felt ready to proceed. If a participant achieved five consecutive correct responses for each color-finger pairing, the screen displayed the message “You have completed the color-response association phase,” and the procedure advanced to the testing phase immediately.

In the testing phase, the repetition effect was assessed by calculating the difference in RT between repeated and non-repeated pairs. The testing phase consisted of two blocks, each containing six practice prime-probe pairs to familiarize participants with the procedure and 36 formal prime-probe pairs. One-third of the pairs featured repeated colored boxes, while two-thirds featured non-repeated ones. Participants were introduced to the trial-pair procedure at the beginning of the testing phase and completed six practice trial-pairs. The experimenter ensured participants had a clear understanding of the experiment after the practice. They had the freedom to commence the formal testing when they felt ready, with approximately 3 mins of rest between the end of the color-response association phase and the formal testing.

At the start of each testing pair, a fixation cross was presented for 300 ms, followed by the appearance of the first colored box. Required responses to the prime box varied based on the session: solely observing the box in the Perception condition, mentally simulating the corresponding keypress while experiencing the virtually recreated movement from a first-person perspective (kinesthetic imagery) in the Imagery condition, and physically pressing the key in the Execution condition. In the Execution condition, the prime box vanished immediately after the keypress or 1800 ms from its onset. In the other two priming conditions, where no actual response was made and, thus, the prime could not be erased at the RT, the duration of the prime presentation was individually adjusted based on the mean RT from the color-response association phase for each colored box. This adjustment was designed to minimize differences in the visible durations of primes across the three priming conditions.

Following the prime response, the screen remained blank for intervals of 75, 150, 225, or 300 ms before the appearance of a fixation cross that preceded the probe box. The probe box was either identical to its preceding prime box (repeated pair) or distinct from it (non-repeated pair). After the 300 ms fixation cross, the probe box was displayed on the screen, and participants were instructed to physically press the corresponding key in all conditions. The probe box disappeared immediately following the keypress or after 1800 ms if no response was registered. Following the presentation of the prime-probe pair, the screen went blank for 75, 150, 225, or 300 ms, followed by a 300 ms fixation cross. A white box then appeared at the center of the screen until participants pressed the key corresponding to their left little finger. Notably, no visual feedback was provided to indicate the accuracy after the key pressing during whole testing phase.

There were 36 trial-pairs in each testing block. After completing each block, participants provided feedback regarding their experience with the prime box on a 7-point Likert scale. In the Perception condition, participants reported their level of focus during the presentation of the prime box. In the Imagery condition, participants assessed the vividness of their motor imagery. In the Execution condition, participants indicated their confidence in making accurate responses. Following the entire experiment, participants also filled out the Chinese version of the Movement Imagery Questionnaire-Revision (cMIQ-R; Hall and Martin, 1997; Lin, 2011).

#### 2.1.5 Data analysis

Probe trials with RTs that that deviated by more than three standard deviations from the mean of each condition for each participant were excluded. This exclusion was carried out using a recursive procedure (Van Selst and Jolicoeur, 1994). Additionally, probe RTs were excluded if the participant responded to the preceding prime in the Perception and Imagery conditions.

The remaining probe RTs were subjected to a two-way repeated-measures ANOVA with factors of Repetition (repeated and non-repeated) and Priming (Perception, Imagery, and Execution). To control for multiple comparisons, *p*-values from *post-hoc* pairwise comparisons were corrected using the step-down method with the Holm-Bonferroni adjustment (Holm, 1979).

To compare the repetition effect between the Imagery and Execution conditions, the differences in RT between non-repeated and repeated probes in these two conditions were calculated and analyzed using paired *t*-tests. Effect sizes were reported as generalized eta square (*η^2^_G_*) for *F*-test and Hedge’s *g* for *t*-test.

To explore potential associations between the repetition effect in Execution (ME), MI ability, and the vividness of MI with the repetition effect in Imagery (MI), Spearman’s rank correlation coefficients were calculated. Specifically, correlations were examined between the repetition effects of Imagery and Execution across participants, as well as between the repetition effect of Imagery and various factors, including the kinesthetic imagery score on the cMIQ-R, visual imagery score on the cMIQ-R, the vividness of Imagery, and the adjusted vividness of Imagery (which accounts for individual differences in confidence levels). To prepare the equivalent sample set for correlation analysis after removing outliers, we identified multivariate outliers by calculating the Mahalanobis distance using all variables included in the correlation analysis. Any data points with Mahalanobis distances exceeding the 95% chi-square cutoff value were subsequently excluded from the analysis.

### 2.2 Results

#### 2.2.1 RT results

**Figure 2:**
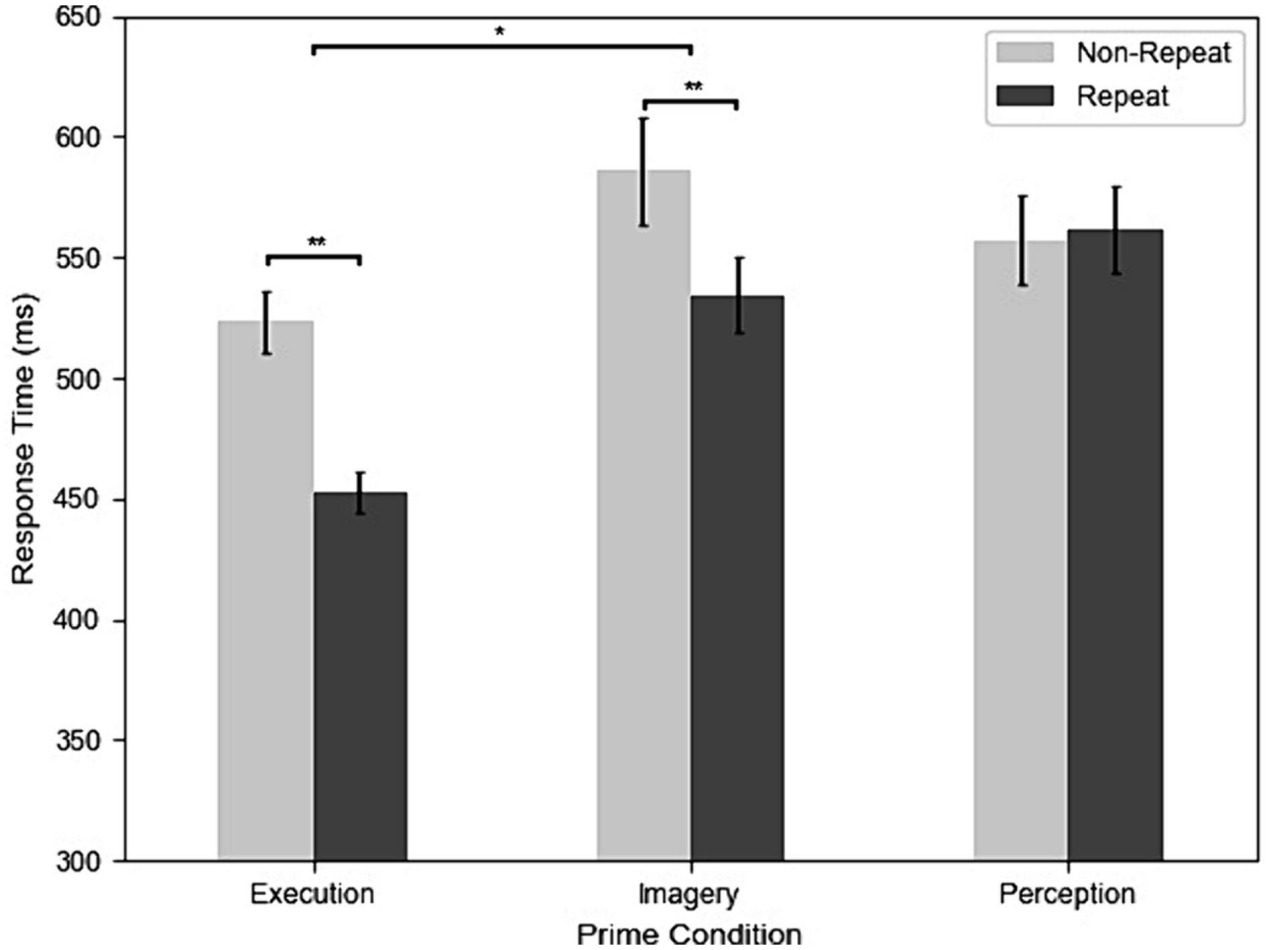
The results of response time (RT) across execution, imagery, and perception in repeated and non-repeated conditions. Error bars indicate the standard errors of each condition (*p < 0.05; **p < 0.01).

Figure 2 shows the RTs of repetition and three different prime conditions from all participants and trials. The results of the two-way repeated-measures ANOVA revealed significant main effects and interactions. The main effect of Priming was significant, *F*(2, 98) = 27.95, *MSE* = 0.11, *p < 0.*001, *η^2^_G_* = 0.12. *Post-hoc* analyses indicated that participants had faster RT in the Execution (499 ms) than the Perception (558 ms) condition, *t(31)* = −6.43, *p < 0.*001, *g* = −0.86, and Imagery (566 ms) condition, *t*(31) = −5.57, *p < 0.*001, *g* = −0.85, but the Imagery and Perception conditions were not significantly different, *t*(31) = 0.11, *p = 0.*916. The main effect of repetition was significant, *F*(1, 31) = 27.43, *MSE* = 0.07, *p < 0.*001, *η^2^_G_* = 0.04, indicating that participants had faster RT in the repeat (515 ms) than the non-repeat (553 ms) condition. The two-way interaction was significant, *F*(2, 98) = 27.39, *MSE* = 0.02, *p < 0.*001, *η^2^_G_* = 0.03. *Post-hoc* analyses indicated that participants had faster RT in the repeat than non-repeat in the Execution condition, *t*(31) = 8.01, *p < 0.*001, *g* = 1.11, and Imagery condition, *t*(31) = 4.55, *p < 0.*001, *g* = 0.47, but not in the Perception condition, *t*(31) = −0.51, *p = 0.*611. In addition, the effect of repetition is larger in the Execution (70 ms) than the Imagery condition (52 ms), *t*(31) = 2.32, *p = 0.*027, *g* = 0.33.

#### 2.2.2 Accuracies

Table 1 shows the results of accuracies. The results of the ANOVA revealed a significant main effect of priming condition on accuracy, *F*(2, 98) = 5.95, *MSE* = 0.01, *p = 0.*005, *η^2^_G_* = 0.05, indicating that participants’ accuracies were lower in Perception (95.07%) than the Imagery (97.26%), *t*(31) = −3.01, *p = 0.*015, *g* = −0.60, and the Execution (96.89%), *t*(31) = −2.60, *p = 0.*028, *g* = −0.49, but not between Imagery and Execution, *t*(31) = 0.62, *p = 0.*539. However, there was no main effect of repetition, *F*(1, 31) = 0.02, *MSE* = 0.00, *p = 0.*877, and no interaction, *F*(2, 98) = 2.05, *MSE* = 0.00, *p = 0.*148.

**Table 1:** Accuracies across execution, imagery, and perception in repeated and non-repeated conditions.

	Perception	Imagery	Execution
Repeated	94.3% (7.3%)	97.4% (4.1%)	97.6% (3.4%)
Non-repeated	95.8% (3.2%)	97.1% (3.3%)	96.2% (3.5%)

#### 2.2.3 Correlations among subjective vividness of MI and RT measures

When examining the correlations between subjective vividness and the repetition effect of Imagery, we introduced individual adjustments to the vividness ratings. This adjustment was based on each participant’s self-estimated accuracy in the Execution condition. This approach aimed to address concerns related to individual differences in confidence when assigning subjective ratings. It is reasonable to assume that individuals who tend to either overrate or underrate their own performance in explicit tasks may similarly exhibit biases in evaluating the subjective vividness of their mental imagery experiences. Therefore, we calculated a confidence weight (CW) as the ratio between real accuracy and subjective accuracy to adjust the tendency in subjective ratings. Subsequently, we multiplied each participant’s subjective ratings by their own CW to derive “adjusted vividness,” which accounted for individual differences in aligning subjective ratings with observed performance.

After reviewing the data and identifying outliers using the multivariate Mahalanobis distance, we excluded two outliers before proceeding with the correlation analysis. The remaining dataset revealed the following correlations: the repetition effect of Imagery was positively correlated with repetition effect of Execution, *r* = 0.60, *p* < 0.001. However, the repetition effect of Imagery was not significantly correlated with the vividness, *r* = 0.01, *p* = 0.978, the adjusted vividness, *r* = 0.05, *p* = 0.783, or the visual imagery score of cMIQ-R, *r* = −0.19, *p* = 0.319, but was marginally correlated with kinesthetic imagery score of cMIQ-R, *r* = −0.36, *p* = 0.052.

### 2.3 Discussion

#### 2.3.1 Repetition effect in MI

The presence of a significant repetition effect in the Imagery condition, but not in the Perception condition, highlights a crucial finding: mere stimulus repetition, without the engagement of actions through either physical execution or mental imagery, is insufficient to elicit the repetition effect. Importantly, the quicker RTs in repeat trials are not attributed to lower accuracy when compared to the RTs in non-repeat trials. In other words, the speed-accuracy trade-off cannot account for the more efficient response following the retrieval of the same S-R binding. This observation underscores the importance of S-R binding as a critical factor contributing to the repetition effect, in line with previous research (Pashler and Baylis, 1991; Henson et al., 2014).

Furthermore, the significant repetition effect observed during mental imagery, where muscle activations are not typically involved, suggests a central origin for this effect (Smith, 1968). This indicates that the repetition effect can arise from mental simulation alone, without physical motor activity.

Moreover, the positive correlation between the repetition effects of MI and ME lends support to the notion that these two processes are related. However, it is important to note that the absence of EMG monitoring in the current experiment raises the possibility that some MI trials may have involved excessive muscle activities, potentially contributing to the observed repetition effect. This concern will be addressed and further clarified in Experiment 2.

#### 2.3.2 Weaker repetition effect in MI than ME

Contrary to the expected outcome, MI showed a weaker repetition effect than ME. Several factors may contribute to the observed weaker repetition effect in the imagery condition: First, participants may engage in response suppression during mental imagery (Guillot et al., 2012), and this inhibitory process could potentially slow down the processing of subsequent responses or stimuli, thereby weakening the repetition effect (Rieger et al., 2017). Second, some participants reported during *post-hoc* interviews that, in the MI condition, they occasionally felt that the stimulus duration estimated from the color-response association phase was too short for them to complete the entire mental imagery process. It is plausible that mental imagery of rapid movements may require more time than its corresponding motor execution (Calmels et al., 2006), and insufficient programming time could impede the full development of the repetition effect in MI. Finally, ME involves both central planning and peripheral muscle activation. These two mechanisms may work in tandem to influence subsequent stimulus–response binding, potentially resulting in a stronger repetition effect.

## 3 Experiment 2: prolonged motor programming period and enhanced control with EMG recording

In Experiment 2, we intend to address a potential confounding factor associated with insufficient programming duration during the Imagery condition. This factor may have contributed to the observed weakening of the repetition effect in comparison to ME and thus affects how conclusion regarding the relationship between MI and ME are drawn. To mitigate this concern, we plan to extend the duration of prime responses in Experiment 2, thereby ensuring that MI can be programmed more comprehensively. Furthermore, in an effort to provide additional clarity and to verify that the repetition effect observed in MI during Experiment 1 was not influenced by subthreshold muscle contractions, we will implement a per-trial monitoring of EMG activity to assess muscle activation in Experiment 2. These methodological refinements will enable us to attain a more robust and comprehensive understanding of the distinct roles of MI and ME in motor repetition effects within the clear definition of MI in our study.

Hétu et al. (2013) have suggested that the occurrence of muscle contractions during imagined movements could introduce variability into neuroimaging responses. Therefore, EMG recording serves as a reliable measure to assess muscle activation during MI. By excluding trials in the Imagery prime condition that exhibited muscle activation in the EMG recordings, we aimed to uncover the repetition effect associated with clear MI which stemming from central processing without even tensing the muscle.

The extension of the prime response duration was crucial to ensure that participants had sufficient time for complete motor programming during MI. In Experiment 1, the brevity of the prime response duration may have limited the extent of motor planning in MI, potentially affecting the observed repetition effect. Therefore, in Experiment 2, we allowed participants more time for mental simulation and motor programming, ensuring that the MI condition was not constrained by time limitations.

The hypothesis driving Experiment 2 posited that if the repetition effect in MI is primarily a result of central cognitive processes and had been previously attenuated due to limited programming time, then a more pronounced and correlated repetition effect between MI and ME should be observable. The expectation was that these effects would remain consistent even after excluding trials with detectable muscle activity. These methodological enhancements in Experiment 2 were crucial for a deeper and more accurate understanding of the distinct roles and interactions of MI and ME in eliciting the repetition effect, all within the defined scope of our study’s exploration of MI.

### 3.1 Materials and methods

#### 3.1.1 Participants

In Experiment 2, a total of 51 participants were initially recruited. However, one participant had to be excluded from further data analysis due to technical issues with the EMG device during the experiment. Therefore, the completed dataset included 50 participants (24 males), with an age range of 18 to 26 years (M = 21.0 years). These participants were distinct from those who participated in Experiment 1. They were also selected from the student population of National Central University, Taiwan, and underwent screening to ensure the absence of known psychotic disorders or visual perception problems. All participants were right-handed, as evidenced by a mean score of 92.3 ± 12.1 on the Edinburgh Handedness Inventory – Short Form (Veale, 2014).

The study protocol received approval from the Research Ethics Committee of National Taiwan University. Prior to their participation, all individuals were provided with comprehensive explanations of the study procedures, their rights, and the potential risks associated with participation. Afterward, they voluntarily signed an informed consent form to participate in the study.

To estimate the sample size, we used data from the first six participants who were included after the process of EMG exclusion. We used G*Power (Faul et al., 2007) to target an effect size of *Cohen’s d* = 0.54, based on the difference between repeated and non-repeated trials in the MI condition. The ideal power was set at 0.80, and the program estimated that 33 participants would be sufficient to reach an actual power of 0.80.

Following the EMG-trial exclusion process, a group of 33 participants (including 8 males) remained for further analysis. These individuals, ranging in age from 18 to 26 years with an average age of 21.2 years, exhibited a pronounced right-handed inclination, as indicated by a mean handedness score of 94.1 ± 10.5.

#### 3.1.2 Design

The design of Experiment 2 closely mirrored that of Experiment 1, except for the number of trials. To account for expected trial drop-outs during the EMG-trimming process, participants in Experiment 2 completed three blocks of trials for each condition, resulting in a total of 108 trials per condition to ensure an adequate number of trials for analysis.

#### 3.1.3 Task, stimuli, and apparatus

The task and equipment used in Experiment 2 largely replicated those of Experiment 1, with a few notable exceptions: To streamline the response interface and improve temporal precision, we replaced the gaming keyboard with a USB response pad (Black Box ToolKit 1–8 button) boasting a sampling rate of 50,000 Hz and a 25-ms key down duration. This response pad featured eight keys, corresponding to the index fingers, middle fingers, ring fingers, and little fingers of both hands.

Additionally, EMG data were recorded using strategically placed electrodes on the right flexor digitorum superficialis, with the ulna serving as the reference electrode and the medial epicondyle as the ground electrode.

For the first 32 participants, a wireless NeXus-10 device from MindMedia BV, Netherlands, was employed for EMG data collection, featuring a sampling rate of 2048 Hz. The EMG voltage values were captured using the Biotrace+ software, also from MindMedia B.V., Netherlands.

For the remaining participants, we utilized a BIOPAC MP36 machine to acquire the EMG signal. The EMG data was recorded and displayed using BIOPAC Student Lab 4.1 software, with a sampling frequency of 2000 Hz. This change in recording equipment was made to improve the signal-to-noise ratio.

It is important to note that the primary purpose of this EMG recording was for screening rather than for analyzing the EMG signal to generate critical results for the current experiment. Consequently, changing the recording device midway through the experiment could have potentially compromised the quality of screening and subsequently impacted the true positive rate of EMG activation detection.

#### 3.1.4 Procedure

Experiment 2 largely followed the procedural framework of Experiment 1, with a notable alteration: the duration of the prime box was fixed at 800 ms for all priming conditions. This was a departure from Experiment 1, where the duration was either individually estimated (Perception and Imagery conditions) or remained onscreen until a response was executed (Execution condition).

#### 3.1.5 Data analysis

The response exclusion criteria remained consistent with those used in Experiment 1. However, in the data preprocessing for Experiment 2, trials featuring EMG responses during the presentation of the prime boxes in the Perception and Imagery conditions were trimmed from the dataset. This was done to specifically evaluate the impact of muscle activation on the repetition effect of motor imagery.

Furthermore, the dataset without screened EMG responses was also analyzed to allow for comparisons with the results obtained in Experiment 1.

#### 3.1.6 EMG preprocessing and analysis

In Experiment 2, the analysis of EMG signals data signals data underwent a two-fold process involving preprocessing and parameter adjustment to ensure the validity of muscle activation detection within each participant.

##### 3.1.6.1 Signal down-sampling and filtering

Initially, the signals were down-sampled to 512 Hz from the NeXus-10 device recording and 500 Hz from the BIOPAC MP36 recording after applying an anti-aliasing filter. Subsequently, the EMG signals were subjected to band-pass filtering within the frequency range of 30 Hz to 55 Hz to focus on relevant muscle activity. To further refine the data, the power of the signals was computed by squaring them. Finally, a 10-point moving average was applied for signal smoothing, which aided in reducing noise and making the data more suitable for subsequent computing.

##### 3.1.6.2 EMG response onset detection

The detection of EMG response onsets was a critical step in understanding the influence of muscle activation on the repetition effect. To achieve this, several statistics were calculated from baseline. The mean (M), standard deviation (SD), mean of the first derivative (dM), and standard deviation of the first derivative (dSD) were computed from a 200 ms interval before the prime boxes’ presentation, serving as baseline. Then, two thresholds were set based on these statistics, utilizing two parameters: C_1_ and C_2_. If the EMG signal value exceeded M+C1×SD , and if the change in signal value exceeded dM+C2×dSD , the onset of the EMG response was marked. These criteria aimed to capture significant deviations from baseline muscle activity.

##### 3.1.6.3 Optimization of parameters

To ensure the validity of the EMG response detection process, the optimal pair of parameters (C_1_ and C_2_) was determined individually for each participant. A comprehensive grid search approach was employed, with C_1_ values ranging from 10 to 30 (in increments of 0.5) and C_2_ values varying from 5 to 10 (also in increments of 0.5). The performance of each parameters pair was evaluated using 108 prime responses in the Execution condition (or 72 trials in the case of one participant due to a technical issue of trigger sending). Evaluation criteria included the distance between the EMG response onset and key press onset and the accuracy of EMG activation detection.

##### 3.1.6.4 EMG detection accuracy

The performance assessment of parameters pairs centered on the accuracy of EMG activation detection. For each pair (designated as p), the accuracy (A_p_) was computed as the ratio of correctly identified trials to the total number of trials. Correct trials were identified by detecting the EMG response onset before the key press onset. The paramount goal was to identify the parameter set that maximized the accuracy of EMG activation detection during the Execution condition.

##### 3.1.6.5 Distance calculation and loss function

In addition to accuracy, the average distance (D_p_) between the EMG-detected onset and the key press onset was calculated for each parameter pair (p) across all Execution prime trials. To holistically assess parameter performance, a loss function (L) was introduced, combining accuracy and temporal precision, defined as $L\left(C1,C2\right)=D_{p}\times 100^{A_{p}}$ . This loss function provided a quantitative measure of how well a specific pair of parameters balanced accuracy and temporal alignment.

Ultimately, the EMG response detection criterion for each participant was thoughtfully determined by selecting the parameter pair that minimized the loss function within the Execution condition. Once this criterion was established, it was consistently applied to detect EMG responses in both the Perception and Imagery conditions.

##### 3.1.6.6 Remaining participants post EMG-trial exclusion

The selection of participants for the EMG-trial-excluded group was contingent upon the parameters derived from their responses during the Execution condition. During this selection procedure, EMG-detected trials were systematically removed from both the Perception and Imagery conditions for each participant. Importantly, participants were excluded from this group only if the number of trial pairs, following the exclusion of EMG data, fell below eight in either sub-condition (e.g., Imagery-repeat).

The criteria for identifying EMG responses were individually established for each participant, guided by the C_1_ and C_2_ values. Remarkably, the average accuracy of EMG response detection during the Execution condition was 89%.

Participants in this group exhibited a mean of 78.15 remaining trials (with a standard deviation of 19.71) in the Perception condition and a mean of 67.97 remaining trials (with a standard deviation of 25.27) in the Imagery condition.

### 3.2 Results

#### 3.2.1 The dataset without screened EMG responses

##### 3.2.1.1 RTs

**Figure 3:**
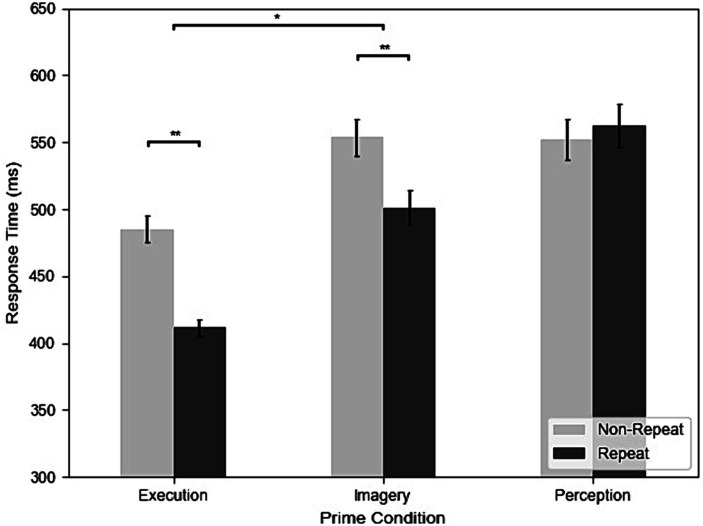
The results of response time (RT) across execution, imagery, and perception from the dataset without screened EMG responses (*p < 0.05; **p < 0.01).

Figure 3 shows the RT of repetition and three different prime conditions from all participants and trials. The results of the RT analysis showed that all main effects and interactions were significant. The main effect of prime condition was significant, *F*(2, 98) = 84.71, *MSE* = 0.30, *p < 0.*001, *η^2^_G_* = 0.25. *Post-hoc* analyses indicated that participants had faster RT in Execution (447 ms) than Perception (554 ms), *t*(49) = −11.29, *p < 0.*001, *g* = −1.45, and Imagery (518 ms), *t*(49) = −9.80, *p < 0.*001, *g* = −1.19 and faster RT in Imagery than Perception, *t*(49) = −4.42, *p < 0.*001, *g* = −0.43. The main effect of repetition was significant, *F*(1, 49) = 67.37, *MSE* = 0.13, *p < 0.*001, *η^2^_G_* = 0.07, indicated that participants had faster RT in repeat (485 ms) than non-repeat (527 ms). The interaction between prime condition and repetition was also significant, *F*(2, 98) = 45.31, *MSE* = 0.04, *p < 0.*001, *η^2^_G_* = 0.05. *Post-hoc* analyses indicated that participants had faster RT in repeat than non-repeat in both Execution, *t*(49) = 10.15, *p < 0.*001, *g* = 1.53, and Imagery, *t*(49) = 6.88, *p < 0.*001, *g* = 0.69. However, this was not the case for the Perception condition, where the difference in RT between repetition and non-repetition was not significant, *t*(49) = −0.75, *p = 0.*455. In addition, the effects of repetition on RT was significantly larger in the Execution condition (77 ms) than the Imagery condition (54 ms), *t*(49) = 3.05, *p = 0.*004, *g* = 0.41.

After removing four outliers using multivariate Mahalanobis distance, we conducted a correlation analysis. The repetition effect of Imagery was positively correlated with repetition effect of Execution, *r* = 0.42, *p = 0.*003, and the vividness, *r* = 0.29, *p* = 0.046. However, the repetition effect of Imagery was not significantly correlated with adjusted vividness, *r* = −0.09, *p* = 0.561, visual imagery score of cMIQ-R, *r* = −0.01, *p* = 0.954, or kinesthetic imagery score of cMIQ-R, *r* = −0. 17, *p* = 0.263.

##### 3.2.1.2 Accuracies

Table 2 shows the results of accuracies. There was a significant main effect of prime condition on accuracy,

**Table 2:** Accuracies across execution, imagery, and perception in repeated and non-repeated conditions in the dataset without screened EMG responses.

	Perception	Imagery	Execution
Repeated	93.1% (6.4%)	98.3% (2.7%)	98.4% (2.2%)
Non-repeated	95.6% (4.6%)	97.1% (3.0%)	96.5% (3.3%)

*F*(2, 98) = 25.12, *MSE* = 0.04, *p* < 0.001, *η^2^_G_* = 0.13. *Post-hoc* analyses indicated that participants had lower accuracy in Perception (94.4%) than the Execution (97.5%), *t*(49) = 4.90, *p* < 0.001, *g* = 0.81, and Imagery (97.7%), *t*(49) = 5.75, *p* < 0.001, *g* = 0.84. The difference in accuracy between Execution and Imagery was not significant, *t*(49) = −0.67, *p* = 0.505. There was no main effect of repetition, *F*(1, 49) = 0.37, *MSE* = 0.00, *p* = 0.544. However, there was a significant interaction, *F*(2, 98) = 18.93, *MSE* = 0.01, *p* < 0.001, *η^2^_G_* = 0.06. *Post-hoc* analyses indicated participants had higher accuracy in repeated pairs than non-repeated pairs in the Execution condition, *t*(49) = −3.25, *p* = 0.004, *g* = −0.67, and in Imagery condition, *t*(49) = −2.48, *p* = 0.016, *g* = −0.43. In the Perception condition, participants had lower accuracy for repeated pairs than non-repeated pairs, *t*(49) = 3.04, *p* = 0.001, *g* = 0.44.

#### 3.2.2 EMG-trial-excluded group

##### 3.2.2.1 RTs

In the analysis of RTs, the main effect of prime condition was found to be significant, *F*(2, 64) = 57.92, *MSE* = 0.21, *p < 0.*001, *η^2^_G_* = 0.29. *Post-hoc* analyses indicated that participants had faster RT in Execution (448 ms) than both the Perception (557 ms), *t*(32) = −9.47, *p < 0.*001, *g* = −1.59, and Imagery (527 ms), *t*(32) = −8.37, *p < 0.*001, *g* = −1.38. RTs in Imagery are also significantly faster than Perception, *t*(32) = −2.89, *p = 0.*007, *g* = −0.37. The main effect of repetition, *F*(1, 32) = 40.20, *MSE* = 0.07, *p < 0.*001, *η^2^_G_* = 0.07, indicated that participants had faster RT in repeat (492 ms) than non-repeat (530 ms). The interaction between prime condition and repetition was also significant, *F*(2, 64) = 28.33, *MSE* = 0.03, *p < 0.*001, *η^2^_G_* = 0.06. *Post-hoc* analyses indicated that participants had faster RT in repeat than non-repeat in both the Execution, *t*(32) = 7.72, *p < 0.*001, *g* = 1.51, and Imagery, *t*(32) = 5.21, *p < 0.*001, *g* = 0.69, but not in the Perception, *t*(32) = −1.38, *p = 0.*176. Furthermore, the repetition effect was significantly larger in the Execution condition (74 ms) than the Imagery condition (52 ms), *t*(32) = 2.24, *p = 0.*032, *g =* 0.38.

In the correlation analysis, none of the correlations were significant, including the repetition effect of Imagery and Execution, *r* = 0.06, *p = 0.*728, and the repetition effect of Imagery was not significantly correlated with measures of vividness, *r* = 0.30, *p* = 0.100, adjusted vividness, *r* = 0.07, *p* = 0.682, visual imagery score of cMIQ-R, *r* = −0.03, *p* = 0.879, or kinesthetic imagery score of cMIQ-R, *r* = −0.26, *p* = 0.160. One outlier was excluded from the analysis using the multivariate Mahalanobis distance.

##### 3.2.2.2 Accuracies

The ANOVA on accuracy revealed a main effect of prime condition, *F*(2, 64) = 11.70, *MSE* = 0.01, *p < 0.*001, *η^2^_G_* = 0.09. *Post-hoc* analyses indicated that participants had lower accuracy in the Perception (95.6%) than the Imagery (98.0%) condition, *t*(32) = 4.38, *p < 0.*001, *g* = 0.68 and Execution (97.7%), *t*(32) = 3.42, *p = 0.*003, *g* = 0.68. Execution and Imagery conditions are not significantly different, *t*(32) = −0.53, *p = 0*.603. There was no significant difference in accuracy between the Execution and Imagery conditions, *F*(1, 32) = 0.08, *MSE* = 0.00, *p = 0.*774. The interaction between prime condition and repetition was significant, *F*(2, 64) = 10.02, *MSE* = 0.01, *p < 0.*001, *η^2^_G_* = 0.05. *Post-hoc* analyses indicated that accuracy had no significant difference in repeated pairs and non-repeated pairs in the Execution condition, *t*(32) = −2.13, *p = 0.*081, and in the Imagery condition, *t*(32) = −0.67, *p = 0*.507, *g* = −0.03. However, participants had lower accuracy in repeated pairs than non-repeated pairs in the Perception condition, *t*(32) = 3.00, *p = 0.*016, *g* = 0.48.

### 3.3 Discussion

In this experiment, we found that even after increasing the duration allowed for performing imagination, the repetition effect in Imagery remained weaker than that observed in Execution. This observation rules out the possibility that the weaker repetition effect in MI time was due to insufficient processing time allocated for mentally simulating motor actions. Moreover, even after excluding trials with excessive EMG activities, the repetition effect still persists in MI. This finding indicates that peripheral factors such as muscle activities cannot account for the repetition effect in MI, which strengthens the evidence for a central origin of repetition effect in MI.

An unexpected outcome here is the lack of significant correlation between the repetition effects of MI and ME. While previous research has often emphasized the overlap between these two modes of action, this result implies that the mechanisms governing the repetition effect in each mode may exhibit certain degree of autonomy. We shall address the possible relationships between MI and ME by considering findings and constraints of both experiments of the current study in the general discussion.

## 4 General discussion

To examine the relationship between MI and ME without reliance on self-report, we compared the repetition effects in both and found it weaker for the former. Furthermore, we also found that extending the duration of the prime stimuli in MI did not enhance its repetition effect but reduced the strength of correlation between the repetition effects in MI and ME. The inequivalent magnitude of repetition effects and the malleable correlation between MI and ME suggest that these two modes of action cannot be entirely equivalent (Jeannerod, 1995; O’Shea and Moran, 2017). The following discussion will consider potential mechanisms involved in the repetition effect and elucidate their roles in MI and ME.

### 4.1 The role of S-R binding in repetition effect

S-R binding is likely the mechanism underlying the repetition effect. However, unlike classical observations of repetition inhibition that are manifested as repetition costs (Rieger et al., 2017; Scheil and Liefooghe, 2018; Bart et al., 2021) we observed consistent repetition facilitation in both MI and ME. This suggests that the shared motor representation between the prime imagery and probe execution may have facilitated repeated responses. Two possible mechanisms for this facilitation are considered here:

First, during MI, the motor programming for responding to the prime stimulus may enhance short-term S-R binding (Ito, 1999; Liefooghe et al., 2021). This enhanced binding could result in faster retrieval when participants encounter the same stimulus again in the probe event, leading to faster responses in the repeated than non-repeated condition. Taken together with the lack of repetition effect in the Perception condition, it is likely that the construction of S-R binding during MI plays a pivotal role in the repetition effect.

Second, the interference from non-repeated prime responses may also contribute to the repetition effect. Some previous studies compared the priming effect of repeated and non-repeated MI with a neutral condition (e.g., rest or imagining both potential responses in prime) and found both the costs associated with alternative actions representation and benefits from repeated actions representation (Li et al., 2005; Ramsey et al., 2010; Toovey et al., 2021). The current findings lend support to the construction of stimulus–response binding during MI rather than counter-response interference as the primary factor contributing to the repetition effect. Specifically, when comparing MI with Perception, imagining actions associated with the prime did not result in slower probe responses than merely perceiving the prime when prime and probe were different, whereas repeated actions during MI prime resulted in faster probe responses than did merely perceiving the prime. The distinction between the Perception and MI conditions ruled out the interference account for the repetition effect.

One can take the probabilities of repetition and the orientation of attention into account when comparing the distinct RT patterns in the repeated vs. non-repeated prime-probe relationship for the Perception and MI conditions. In Toovey et al. (2021), the ratio of repeated to non-repeated pairs was 80:20, an unbalanced ratio that likely led participants to expect the same response after the prime. Consequently, when compared to the neutral condition, the lower predictive ratio hindered the response process in non-repeated probes. Moreover, their experimental design involved stimuli with two spatial orientations (e.g., left and right) corresponding to responses, which may have led to suppression of the opposite side when one’s attention is already oriented toward the other direction (May et al., 1995). Similarly, Li et al. (2005) reported motor interference with MI when participants performed hand flexion and extension in their action priming paradigm. They found that motor interference from prime imagery occurred when the subsequent execution involved the contraction of antagonist muscles. Thus, interference may occur when alternative stimuli or responses in the choice set can induce suppression, either from opposite attention orientations or antagonist muscles.

The current study distinguished stimuli using colors instead of spatial orientation or the agonist–antagonist relationship. This color-based mapping is potentially less intuitive than mappings based on spatial or kinesthetic factors, which might explain the diminished suppression in the non-repeated prime response. This variation could contribute to the contrast in repetition facilitation observed in our study vs. the repetition inhibition reported in earlier research.

### 4.2 Stronger S-R binding in prime execution than prime imagery

Comparing repetition effects in the Imagery and Execution conditions of our study reveals that actual responses to prime stimuli foster more defined and specific motor processes. This leads to a more marked repetition effect than that observed in responses to prime Imagery. This observation is consistent with Toovey et al. (2021) findings which also employed a repetition paradigm to compare MI and MP (motor preparation, namely the motor planning phase before actual execution). Their study found a stronger repetition effect in MI compared to MP, implying that MI encompasses more comprehensive information, including sensory feedback predictions elicited by imagined movements. This detailed information likely forms a complex association with the stimulus, which is then reactivated when participants encounter the same stimulus subsequently. Incorporating this logic into our study, it is plausible to suggest that the genuine sensorimotor information involved in responding to the prime enhances the overlap in motor processes between the prime and the following probe, especially when an action is physically executed for the prime rather than merely imagined. The richer and more specific information obtained from processing and executing the prime likely prepares the effector system for the probe, consequently resulting in a stronger repetition effect.

Incomplete Overlap between the Motor Processes of MI and ME While the finding of repetition benefits in both Imagery and Execution conditions aligns with the notion of functional equivalence, which posits that these two conditions share similar underlying mechanisms, a stronger prediction of this theoretical perspective would entail a significant correlation between the repetition effects observed in these two conditions. In other words, individuals who exhibit a stronger repetition effect in prime Imagery should also demonstrate a correspondingly stronger repetition effect in prime Execution, reflecting the shared mechanisms between the two. However, in the current study, this prediction did not hold when we extended the response time for MI and excluded trials with muscle activation during MI. This discrepancy suggests that the relationship between repetition effects in Imagery and Execution may be more complex and influenced by nuanced factors.

What could be the non-overlapped parts between MI and ME? One possibility is that MI involves a higher degree of awareness and monitoring of motor processes than ME. Jeannerod (2001) proposed that cognitive states of simulated actions can vary along a spectrum of different levels of awareness. In our study, participants could evaluate and report the subjective vividness of their MI experiences, suggesting explicit and deliberate representations of the action. Moreover, previous neuroimaging studies assessing the explicitness, awareness, and attentiveness of MI have highlighted the involvement of the frontal–parietal network (Gerardin et al., 2000; Rushworth et al., 2003; Fridman et al., 2011; Lorey et al., 2011; Vry et al., 2012), which is also considered critical for motor awareness (Desmurget and Sirigu, 2009). The idea of conscious monitoring in MI concurs with the motor-cognitive model (Glover and Baran, 2017; Glover et al., 2020), which posits that MI and ME have distinct real-time control process. According to this framework, both MI and ME share common motor representations during pre-movement planning but diverge during real-time operations. MI requires conscious executive control processes, such as elaboration and monitoring, whereas ME can access online feedback without awareness (Johnson and Haggard, 2005; Cameron et al., 2009). This contrasts with the functional equivalence hypothesis which suggests that MI and ME involve similar mechanisms.

As our study required participants to perform a simple key-pressing response, they may have executed the key-pressing in an “auto-pilot” manner with limited conscious awareness of the movement process. During MI, avoiding deliberate processing awareness is challenging, even in simple key pressing tasks. Conversely, actual response execution can unfold quite automatically, involving minimal attention and awareness of the motor control process. This divergence in conscious awareness might account for the inconsistent correlation results observed between MI and ME in Experiment 1 and Experiment 2. In Experiment 1, the brief prime stimulus presentation may have expedited imagery, diminishing awareness of the imagery process and prompting more subliminal muscle activation. This uncontrolled muscle activation during MI leads to a similar processing pattern with ME. In contrast, the prolonged prime duration in Experiment 2 facilitated a smoother mental simulation of movements, allowing ample time to elaborate and monitor the motor control process during imagery. With controlled EMG activation, Experiment 2 indicates that the repetition effect in MI originates more purely from a central and top-down source, distinguishing it from ME. Our results align more closely with the motor-cognitive model, positing distinct online operations for Motor Imagery and Motor Execution, than with the functional equivalence hypothesis. We recommend that future research comparing MI and ME should evaluate not only their differences but also their covariation. While differences in MI and ME measures can highlight their dissimilarities, examining covariation can provide further understanding of the degree of overlap in their underlying processes.

### 4.3 Limitations

#### 4.3.1 Accuracy of MI

Several limitations in the current study should be noted. MI is inherently a private and subjective process, and errors in MI can lead to trials being mistakenly categorized as repeated or non-repeated. Accurately measuring MI trial-by-trial without interfering with the task (e.g., requiring participants to report their accuracy after each trial) can introduce noise into the assessment of repetition effects in MI. To maintain equivalence across conditions, ME trials with incorrect responses to primes were not excluded from the analysis. However, given the high accuracies observed across participants in the ME condition, we believe the inferences drawn in this study remain valid.

#### 4.3.2 Stimulus-driven vs. intention-driven action

In contrast to most studies on MI that have adopted subjective duration measurements, the current study generalizes the relationship between MI and ME in the simple visuomotor responses, which can be considered a type of stimulus-driven action (Herwig et al., 2007; Herwig and Waszak, 2012). Whether the conclusions regarding functional equivalence derived from this study extend to more complex, intention-driven types of movements remain to be clarified. Future research on after-effects of intention-based action (e.g., spontaneously selected key pressing) may offer valuable insights into the relationship between MI and ME in such contexts.

#### 4.3.3 Incomparable response transition between MI and ME

In our study, participants alternated between responding with imagery and execution in the MI conditions, and between simply observing and executing in the Perception condition, but not in the ME condition. This mode-switching design might have contributed to the primary effect observed in the prime condition, where responses to the probe were faster in the ME condition compared to the MI and Perception conditions, regardless of repetition. Previous research has demonstrated that the binding between stimuli and responses can facilitate responses within the same mode but hinder switching between modes (Rieger et al., 2017; Scheil and Liefooghe, 2018). In their experiment, response times in MI were directly measured through motor responses initiated at the onset of MI, and they found that the same action could be facilitated when executed in the same response mode (e.g., ME-ME or MI-MI) but disrupted when switching between different response modes (e.g., MI-ME or ME-MI). However, our study aimed to investigate pure MI without requiring participants to indicate the onset of MI through indirect measures. This design aimed to prevent contamination of the motor representation of MI by pre- and post-motor responses. Future studies examining the differential magnitude of repetition effects between prime imagery and prime execution should consider the potential impact of mode-switching on the repetition effect and develop innovative methods to assess this influence.

#### 4.3.4 Impacts of physical execution on imagery

Moreover, a notable aspect of our experimental design was the deliberate exclusion of “Imagery-Imagery” and “Execution-Imagery” pairs. This decision was primarily guided by the underlying logic of the repetition effect, which is central to our study. The repetition effect, as observed in motor control studies, typically manifests when the same action or task is repeated, leading to enhanced performance due to factors like priming, increased familiarity, and neural efficiency. In our experiments, we focused on “Imagery-Execution” and “Execution-Execution” pairs to directly assess this effect.

The “Imagery-Imagery” pair was excluded because repeating an imagined action without interspersing it with a physical execution would not have provided the contrast necessary to explore the primary aim of our study, which is to investigate the functional equivalence and relationship between MI and ME. Moreover, the repetition of imagery alone would likely fall short in demonstrating the cognitive and neural overlap between MI and ME, as our interest was in examining how imagined actions influence subsequent executed actions and vice versa.

Our study intentionally did not include the “Execution-Imagery” pairing as it diverges from our main objective, which was to explore how prior mental simulation (imagery) influences subsequent physical execution. Incorporating “Execution-Imagery” would have shifted the focus toward how physical execution primes mental simulation, deviating from our central theme. However, future research, particularly studies employing EEG/MEG or fMRI techniques, could benefit from including this pairing to investigate the covert neural processes involved, offering a different perspective on the interaction between executed actions and subsequent mental imagery.

## 5 Conclusion

Based on the findings of the current study, we have provided a method that may allow researchers to more objectively assess the impacts of MI on behavioral outcomes. By highlighting differences and the absence of correlation in repetition effects, this study challenges the functional equivalence hypothesis of imagery and execution. Our results suggest that motor representations of imagery and execution, when measured with responses that are not directly linked to the subjective aspects of the mental imagery, are more distinguishable than traditionally thought. The differential repetition effect between motor imagery and execution provide a methodological route for future studies aiming at examining cognitive and neural mechanisms of motor imagery under minimal impacts of subjective experiences.

## Data availability statement

The datasets presented in this study can be found in online repositories. The names of the repository/repositories and accession number(s) can be found at: https://osf.io/b74ac/?view_only=96d7e4c10bac4923a96712980e9fbe31.

## Ethics statement

The studies involving humans were approved by Research Ethics Committee of National Taiwan University. The studies were conducted in accordance with the local legislation and institutional requirements. The participants provided their written informed consent to participate in this study.

## Author contributions

H-PT: Conceptualization, Data curation, Formal analysis, Methodology, Software, Visualization, Writing – original draft, Writing – review & editing. EC: Funding acquisition, Investigation, Methodology, Project administration, Resources, Supervision, Validation, Writing – review & editing.
